# Clinical value of Cyclin D1 and P21 in the differential diagnosis of papillary thyroid carcinoma

**DOI:** 10.1186/s13000-023-01410-z

**Published:** 2023-11-11

**Authors:** Chen-chen Wang, Dan-dan Lu, Ming-hong Shen, Ru-lei Chen, Zhi-hong Zhang, Jing-huan Lv

**Affiliations:** 1 Department of Pathology, Gusu School, the Affiliated Suzhou Hospital of Nanjing Medical University, Suzhou Municipal Hospital, Nanjing Medical University, Suzhou, China; 2 Department of Pathology, the First Affiliated Hospital of Nanjing Medical University, Nanjing, China

**Keywords:** Papillary thyroid carcinoma, Cyclin D1, P21, Thyroid tissue in cervical lymph nodes

## Abstract

**Background:**

With the continuous discovery of new borderline thyroid lesions and benign and malignant “gray areas”, coupled with the limitations of traditional immune indicators, the differential diagnosis of papillary thyroid carcinoma (PTC) has become more difficult. Cyclin D1 and P21 are cell cycle regulators involved in the occurrence and metastasis of multiple tumors, including PTC, but their specific functions are unclear.

**Methods:**

In our study, immunohistochemical staining was used to explore the expression of Cyclin D1 and P21 in PTC, paracancerous tissue, follicular adenoma (FA) and papillary thyroid hyperplasia. In addition, their relationship with the clinicopathological features of PTC and their differential diagnostic value in distinguishing between intralymph node PTC metastases and intralymph node ectopic thyroid tissue were studied.

**Results:**

Among 200 primary PTC lesions, Cyclin D1 and P21 were found to be expressed in 186 (93.00%) and 177 (88.50%), respectively, and their expression levels were significantly higher in PTC tissue than in adjacent tissue, FA tissue and papillary thyroid hyperplasia tissue (*P* < 0.05). The expression levels of Cyclin D1 and P21 were positively correlated with tumor size and lymph node metastasis (*P* < 0.05) but not with sex, age, number of tumor lesions, histological subtype, chronic lymphocytic thyroiditis or TNM stage (*P* < 0.05). The expression levels of Cyclin D1 and P21 were significantly correlated (*P* < 0.05). The positivity rates of Cyclin D1 and P21 in intralymph node PTC metastases were 97.96% (48/49) and 89.80% (44/49), respectively, which were significantly higher than those in intralymph node ectopic thyroid tissue (*P* < 0.05). The sensitivity (Se) and negative predictive value (NPV) of Cyclin D1 and P21 detection alone or in combination were higher than those of the combined detection of the classical antibody markers CK19, HBME-1 and Galectin-3. Besides, the Se, Sp, PPV and NPV of Cyclin D1 and P21 in differentiating intralymph node PTC metastases and intralymph node ectopic thyroid tissue were higher.

**Conclusions:**

The results of our study show that Cyclin D1 and P21 are highly sensitive and specific markers for the diagnosis of PTC that are superior to traditional classical antibodies. And, these two markers are of great value in the differential diagnosis of intralymph node PTC metastases and intralymph node ectopic thyroid tissue.

## Introduction

Papillary thyroid carcinoma (PTC) is the most common type of thyroid cancer [1]. It can be divided based on its histological morphology into the classic type, follicular variant, encapsulated variant, oncocytic variant, columnar cell variant and other variants, but differential diagnosis is more difficult. At the same time, regarding clinicopathological diagnosis, the diameter of papillary microcarcinoma is sometimes less than 1 mm, and it can exhibit only a follicular structure, a lack of interstitial infiltration, or interstitial sclerosis, so the diagnosis is difficult. Other types, such as follicular variants of papillary thyroid carcinoma (FVPTC) and follicular adenoma (FA) and cervical lymph node papillary carcinoma metastasis and ectopic thyroid tissue, are all difficult to diagnose. Thus, identifying reliable markers has important practical clinical application value.

Cyclin D1 belongs to the cyclin family and is encoded by the CCND1 gene located on chromosome 11q13. P21 belongs to the cyclin-dependent kinase inhibitor (CDKI) family and is located on the short arm of chromosome VI downstream of the P53 gene. Cyclin D1 and P21, with cyclin-dependent kinase (CDK) as the core, jointly regulate the progression of the cell cycle from G1 phase to S phase. The abnormal expression of P21 protein affects the activity of cyclin, CDKs and their kinases, thereby affecting cell proliferation and differentiation and ultimately leading to the development of tumors [2]. Early studies have found that the expression of Cyclin D1 is related to the degree of differentiation of papillary thyroid carcinoma, its invasive biological behavior and the presence of lymph node metastasis [3]. In addition, studies have found that Cyclin D1 is a molecular marker for the dedifferentiation of papillary thyroid carcinoma into anaplastic papillary carcinoma, indicating an imbalance in cell cycle control and increased proliferative activity in anaplastic papillary carcinoma [4]. Previous studies have shown that P21, as a cell cycle regulatory protein, plays an important role in different types of tumors with different functions [5]. However, there is no relevant research on the expression of P21 in thyroid tumors, and there is a lack of human histological data.

Therefore, in this study, we collected human PTC, adjacent tissues, FA, and papillary thyroid hyperplasia to detect the expression of Cyclin D1 and P21 proteins and to determine their relationship with the clinicopathological characteristics of PTC in order to provide reliable auxiliary indicators for difficult cases of PTC.

## Material and methods

### Case selection

Surgically resected PTC, FA and follicular epithelial papillary hyperplasia specimens were collected from January 2016 to December 2021 at Suzhou Hospital affiliated with Nanjing Medical University. Corresponding lymph node dissection specimens and matched normal adjacent tissue were also collected. All patients had no history of radiotherapy or chemotherapy before surgery. The adjacent tissue was taken from normal thyroid tissue around the PTC foci at a distance of 3 cm. All cases were reviewed for diagnosis by senior diagnosticians at the pathology department of our hospital.

There were 200 cases of papillary thyroid carcinoma and adjacent tissues, including 53 males and 147 females. The age range was 14–82 years (median age, 48 years), 166 cases were < 55 years old, and 34 cases were ≥ 55 years old. There were 14 cases with a maximum tumor diameter < 0.5 cm, 92 cases with ≥ 0.5, ≤ 1 cm, 69 cases with > 1, ≤ 2 cm, and 25 cases with > 2 cm. A total of 138 cases were unilateral, and 62 cases were multiple. There were 185 cases of classic PTC, and 15 cases of the follicular variant. Forty-one cases were complicated with chronic lymphocytic thyroiditis, and 159 cases were not. Lymphatic metastases were observed in 117 cases but not the other 83 cases. In terms of TNM staging, there were 196 cases of stage I/II and 4 cases of stage III/IV.

There were 39 cases of follicular adenoma in 10 males and 29 females, and the age range was 24–77 years (median age, 44 years). There were 40 cases of papillary hyperplasia of the thyroid follicular epithelium in 7 males and 33 females, and the age range was 32–82 years (median age, 58 years). There were 49 cases of intralymph node PTC metastases and 15 cases of intralymph node ectopic thyroid tissue.

### Immunohistochemical staining

All specimens were fixed in 10% neutral formalin and embedded in paraffin. The primary antibodies against CK19, HBME-1, and Galectin-3 were purchased from Beijing Zhongshan Golden Bridge Company, and primary antibodies against Cyclin D1 and P21 were purchased from Gene Tech (Shanghai) Company Limited A Gene Group Company. Immunohistochemical staining was performed by the En Vision method. The experimental procedure was performed in strict accordance with the instructions.

The immunohistochemistry results showed CK19, HBME-1, and Galectin-3 positivity in the cytoplasm, and positive staining appeared brown or yellow. Cyclin D1 and P21 positivity was observed in the nucleus, which was comprehensively interpreted according to the staining intensity and the proportion of stained areas. Nuclear intensity was scored as follows: 0 = negative, 1 = yellow (weak), 2 = brown (moderate), and 3 = tan (strong). The proportion of the stained area was interpreted as the ratio of positive cells to the total number of tumor cells, and the estimated fractions were denoted as 0 (0–1%), 1 (2–25%), 2 (26–50%), 3 (51–75%), and 4 (> 75%). A combined nuclear score (NS) was constructed according to the intensity and multiplying fraction. A P21 final score < 2 was considered negative expression, a score of 2–5 was considered moderate expression, and ≥ 6 was considered high expression. A Cyclin D1 final score ≤ 2 was considered negative expression, 3–5 was considered moderate expression, and ≥ 6 was considered high expression.

### Statistical analysis

All statistical analyses were carried out using IBM SPSS Statistics for Windows, version 20 (IBM Corp., N.Y., USA). The expression difference in Cyclin D1 and P21 among the different groups was analyzed with the Fisher exact test or χ2 test. The correlation between Cyclin D1 and P21 expression and different clinicopathological variables was analyzed using Fisher exact test or the χ2 test. Correlations among the expression levels of Cyclin D1 and P21 were evaluated by Spearman’s bivariate correlation test. Differences were considered statistically significant at *P* < 0.05.

## Results

### Expression of Cyclin D1 protein increased in primary PTC lesions and its correlation with clinicopathological features

**Fig. 1:**
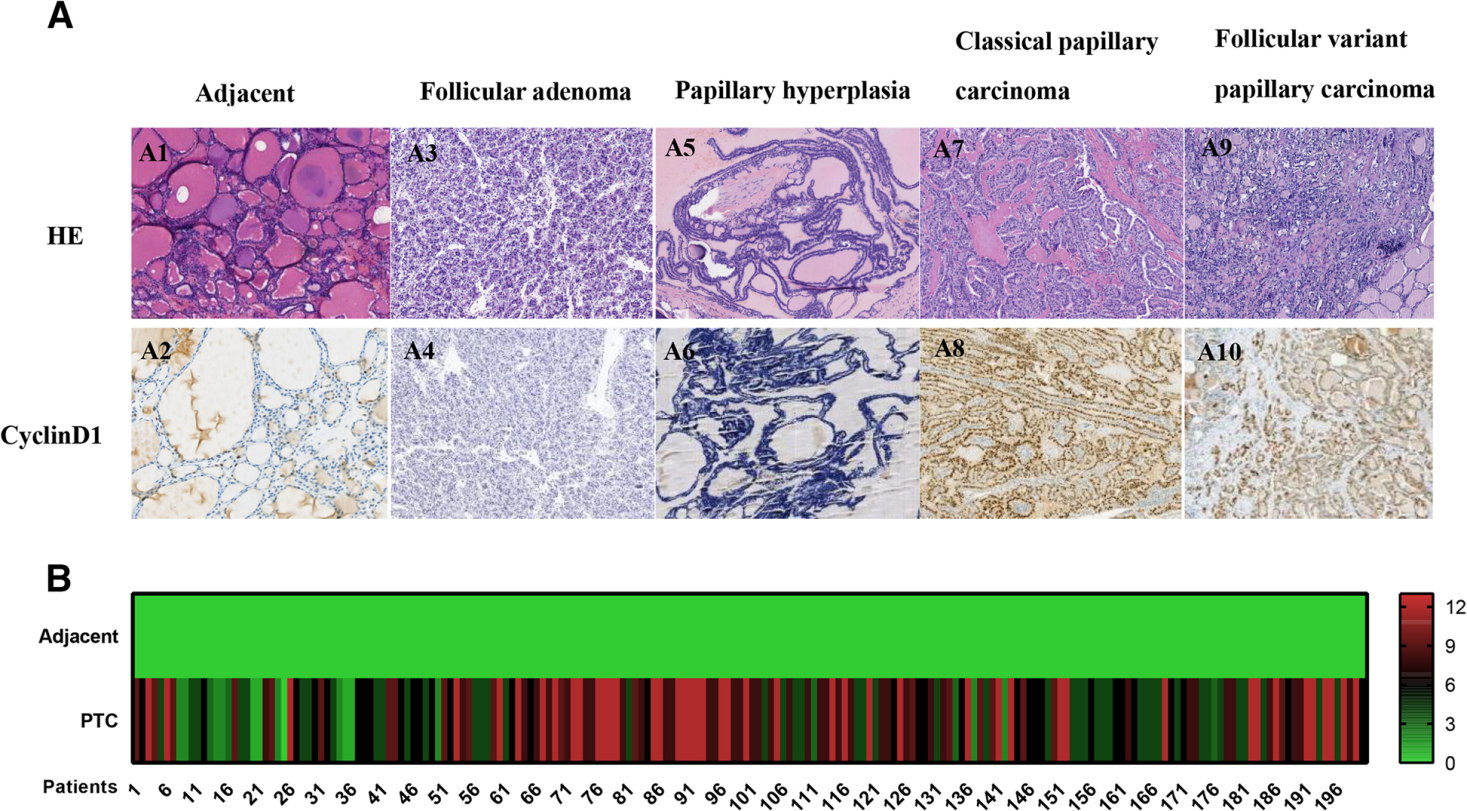
**A** Histological morphology (100 ×) and Cyclin D1 immunohistochemical staining (100 ×) of adjacent, follicular adenoma, thyroid follicular papillary hyperplasia, classical papillary carcinoma and follicular variant papillary thyroid carcinoma. A1. Histologic features of adjacent tissue; A2. Adjacent tissue does not express Cyclin D1; A3. Histologic features of follicular adenoma; A4. Follicular adenoma does not express Cyclin D1;A5. Histologic features of thyroid follicular papillary hyperplasia; A6. Thyroid follicular papillary hyperplasia does not express Cyclin D1; A7. Histologic features of classical papillary thyroid carcinoma; A8. Positive Cyclin D1 expression in classical papillary carcinoma; A9. Histologic features of follicular variant papillary thyroid carcinoma; A10. Cyclin D1 positive expression in follicular variant papillary thyroid carcinoma. **B** Immunohistochemical scoring of Cyclin D1 protein in 200 paired adjacent and papillary thyroid carcinomas

Cyclin D1 protein expression is localized in the nucleus, and adjacent thyroid tissue does not express Cyclin D1 (Fig. 1A). The positive expression rates of Cyclin D1 in PTC, FA and follicular papillary hyperplasia were 93.00% (186/200), 25.64% (10/39), and 2.50% (1/40), respectively (Fig. 1B and Table 1). The expression of Cyclin D1 protein in PTC was significantly higher than that in the paired adjacent tissue (*P* < 0.05, Table 1) and higher than that in the FA and follicular papillary hyperplasia, showing a significant difference (*P* < 0.05, Table 1).

**Table 1:** Difference in Cyclin D1 protein and P21 protein expression among the four groups

Group	n	Cyclin D1			χ2 test	comparison of Cyclin D1 positive rates		
		Positive	Negative	Positive rate(%)		PTC VS Adjacent	PTC VS FA	PTC VS PH
Adjacent	200	0	200	0.00	χ2	347.66	12.55	128.67
Papillary thyroid carcinoma	200	186	14	93.00				
Follicular adenoma	39	10	29	25.64	*P*	0.000	0.000	0.000
Papillary hyperplasia	40	1	39	2.50				
Group	n	P21			χ2 test	comparison of P21 positive rates		
		Positive	Negative	Positive rate(%)		PTC VS Adjacent	PTC VS FA	PTC VS PH
Adjacent	200	0	200	0.00	χ2	317.49	120.65	134.86
Papillary thyroid carcinoma	200	177	23	88.50				
Follicular adenoma	39	2	37	5.13	*P*	0.000	0.000	0.000
Papillary hyperplasia	40	0	40	0.00				

*PTC* Papillary thyroid carcinoma, *FA* Follicular adenoma, *PH* Papillary hyperplasia

Among the 200 cases of PTC, 7.00% (14/200) were negative for Cyclin D1, 25.50% (51/200) had moderate expression, and 67.50% (135/200) had high expression (Table 2). Cyclin D1 protein expression was positively correlated with tumor size (*P* < 0.05) and lymph node metastasis (*P* < 0.05), but was not significantly correlated with patient sex, age, number of tumor nests, histological subtype, the presence or absence of chronic lymphocytic thyroiditis or TNM stage (Table 2).

**Table 2:** Correlation between Cyclin D1 and P21 expression in primary PTC lesions and clinicopathological variables

Clinicopathological factors	n	Cyclin D1 expression(%)					P21 expression(%)				
		Negative	Intermediate	High	χ2	*P-value*	Negative	Intermediate	High	χ2	*P-value*
Gender					3.075	0.215				1.968	0.374
Male	56	3(5.36)	10(17.86)	43(76.78)			4(7.14)	28(50.00)	24(42.86)		
Female	144	11(7.64)	41(28.47)	92(63.89)			19(13.19)	60(41.67)	65(45.14)		
Age (years)					0.366	0.833				1.970	0.373
< 55	166	12(7.23)	41(24.70)	113(68.07)			21(12.65)	70(42.17)	75(45.18)		
≥ 55	34	2(5.88)	10(29.41)	22(64.71)			2(5.88)	18(52.94)	14(41.18)		
Primary tumor size (cm)					-^a^	0.000				41.014	0.000
< 0.5	14	3(21.43)	7(50.00)	4(28.57)			4(28.57)	8(57.14)	2(14.29)		
≥ 0.5, ≤ 1	92	8(8.69)	33(35.87)	51(55.43)			15(16.30)	53(57.61)	24(26.09)		
> 1, ≤ 2	69	3(4.35)	9(13.04)	57(82.61)			4(5.80)	21(30.43)	44(63.77)		
> 2	25	0(0.00)	2(8.00)	23(92.00)			0(0.00)	6(24.00)	19(76.00)		
Number of tumors					2.232	0.328				0.296	0.862
Single	138	12(8.70)	36(26.09)	90(65.22)			17(12.32)	60(43.48)	61(44.20)		
multiple	62	2(3.23)	15(24.19)	45(72.81)			6(9.68)	28(45.16)	28(45.16)		
Histological subtype					-^a^	0.104				4.429	0.109
Classic	185	14(7.57)	44(23.78)	127(68.65)			19(10.27)	81(43.78)	85(45.95)		
Follicular variant	15	0(0.00)	7(46.67)	8(53.33)			4(26.67)	7(46.67)	4(26.67)		
Chronic lymphocytic thyroiditis					0.372	0.830				0.950	0.622
Absent	159	12(7.55)	40(25.16)	107(67.30)			19(11.95)	72(45.28)	68(42.77)		
Present	41	2(4.88)	11(26.83)	28(68.29)			4(9.76)	16(39.02)	21(51.22)		
Lymph node metastasis					22.764	0.000				8.266	0.016
Absent	83	7(8.43)	35(42.17)	41(49.40)			12(14.46)	44(53.01)	27(32.53)		
Present	117	7(5.98)	16(13.68)	94(80.34)			11(9.40)	44(37.61)	62(52.99)		
TNM					-^a^	0.080				-^a^	0.353
I + II	196	13(6.63)	50(25.51)	133(67.86)			23(11.73)	85(43.37)	88(44.90)		
III + IV	4	0(0.00)	3(75.00)	1(25.00)			1(25.00)	1(25.00)	2(50.00)		

^a^Fisher exact test

### Increased P21 protein expression in primary PTC lesions and its correlation with clinicopathological features

**Fig. 2:**
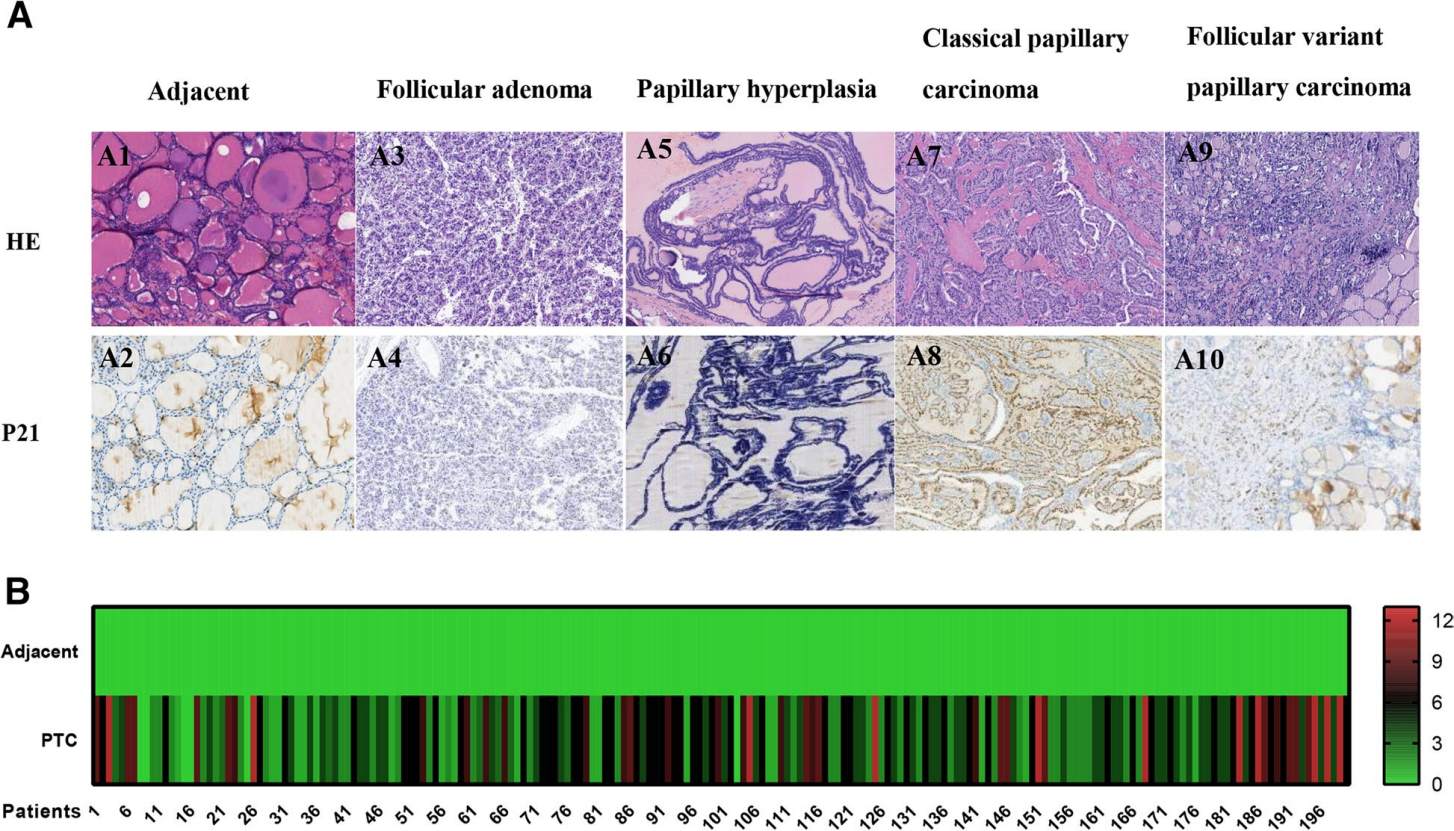
**A** Histological morphology (100 ×) and P21 immunohistochemical staining (100 ×) of adjacent, follicular adenoma, thyroid follicular papillary hyperplasia, classical papillary carcinoma and follicular variant papillary thyroid carcinoma. A1. Histologic features of adjacent; A2. Adjacent does not express P21; A3. Histologic features of follicular adenoma; A4. Follicular adenoma does not express P21; A5. Histologic features of thyroid follicular papillary hyperplasia; A6. Thyroid follicular papillary hyperplasia does not express P21; A7. Histologic features of classical papillary thyroid carcinoma; A8. Positive P21 expression in classical papillary carcinoma; A9. Histologic features of follicular variant papillary thyroid carcinoma; A10. Positive P21 expression in follicular variant papillary thyroid carcinoma. **B** Immunohistochemical scoring of P21 protein in 200 paired adjacent and papillary thyroid carcinomas

P21 protein expression was localized in the nucleus, and adjacent thyroid tissue did not express P21 (Fig. 2A). The proportion of patients with positive expression of P21 among those with PTC, FA and follicular papillary hyperplasia were 88.50% (177/200), 5.13% (2/39) and 0.00% (0/40), respectively (Fig. 2B and Table 1). The expression of P21 protein in PTC tissue was significantly higher than that in the paired adjacent tissue (*P* < 0.05, Table 1) and significantly higher than that in FA and follicular papillary hyperplasia tissues (*P* < 0.05, Table 1).

Among the 200 cases of PTC, 11.50% (23/200) were negative for P21, 44.00% (88/200) had moderate expression, and 44.50% (89/200) had high expression (Table 2). P21 protein expression was positively correlated with tumor size (*P* < 0.05) and lymph node metastasis (*P* < 0.05) but was not correlated with patient sex, age, number of tumor nests, histological subtype, the presence or absence of chronic lymphocytic thyroiditis or TNM stage (Table 2).

### Correlation between Cyclin D1 protein and P21 protein expression in primary PTC lesions.

Among the 200 cases of PTC, the immunophenotype was Cyclin D1-/P21- in 4.00% (8/200), Cyclin D1-/P21 + in 3.00% (6/200), Cyclin D1 + /P21- in 7.50% (15/200), and Cyclin D1 + /P21 + in 85.50% (171/200). There was a significant correlation between Cyclin D1 protein expression and P21 protein expression (*P* < 0.05) (Table 3).

**Table 3:** Correlation between Cyclin D1 and P21 expression in primary PTC lesions

P21 expression(%)	n	Cyclin D1 expression(%)			*r*_*s-*_*value*	*P*_*-*_*value*
		Negative	Intermediate	High		
Negative	23	8 (34.78)	11 (47.83)	4 (17.39)	0.587	0.000
Intermediate	88	5 (5.68)	37 (42.05)	46 (52.27)		
High	89	1 (1.12)	3 (3.37)	85 (95.51)		

### Difference in Cyclin D1 protein expression and P21 protein expression in intralymph node PTC metastases and intralymph node ectopic thyroid tissue.

**Fig. 3:**
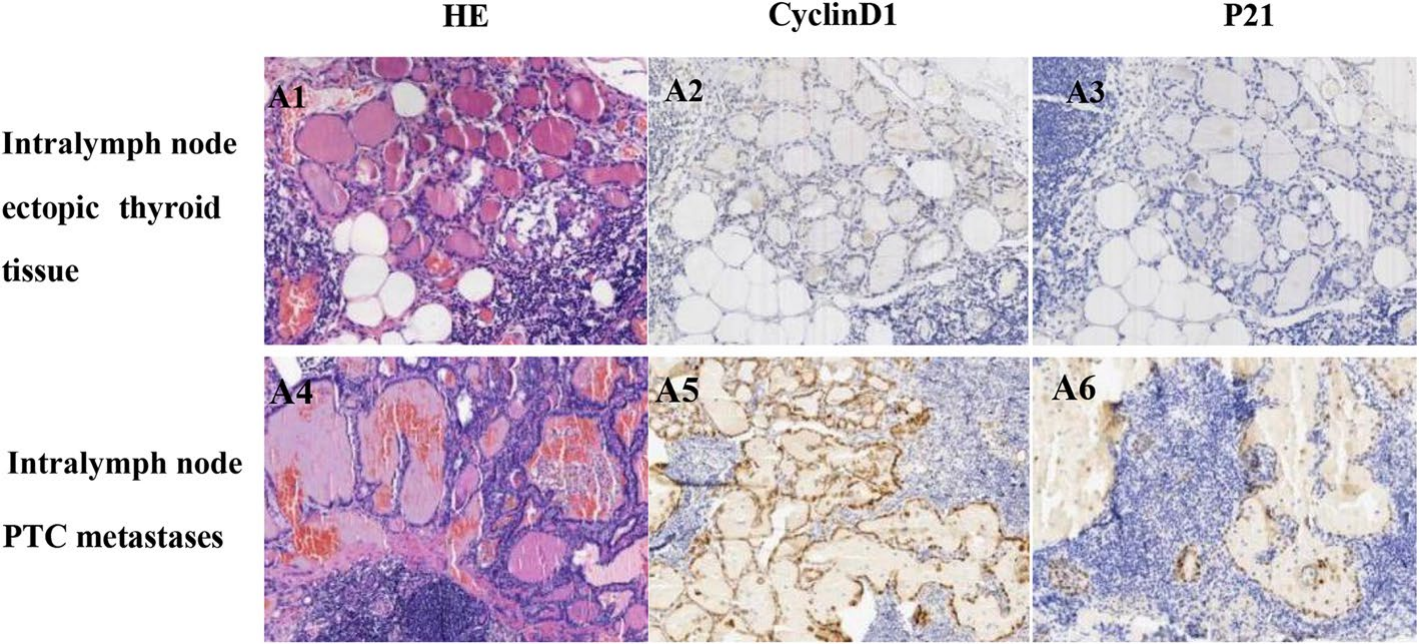
Histological morphology (200 ×) and immunohistochemical (200 ×) expression of Cyclin D1 and P21 in intralymph node ectopic thyroid tissue and intralymph node PTC metastases. A1. Histological morphology of intralymph node ectopic thyroid tissue; A2. Intralymph node ectopic thyroid tissue does not express Cyclin D1; A3. Intralymph node ectopic thyroid tissue nodes do not express P21; A4. Histological morphology of intralymph node PTC metastases; A5. Cyclin D1 positive expression in intralymph node PTC metastases; A6. Positive P21 expression in intralymph node PTC metastases

The positivity rates of Cyclin D1 and P21 in intralymph node PTC metastases were 97.96% (48/49) and 89.80% (44/49), respectively, while those in intralymph node ectopic thyroid tissue were 26.67% (4/11) and 13.33% (2/15), respectively (Fig. 3 and Table 4). The expression levels of Cyclin D1 and P21 were elevated in intralymph node PTC metastases and were significantly different from those in intralymph node ectopic thyroid tissue (*P* < 0.05, Table 4).

**Table 4:** Difference in Cyclin D1 and P21 expression in intralymph node PTC metastases and intralymph node ectopic thyroid tissue

Group	n	CyclinD1			*P-value* ^a^	P21			*P-value* ^a^
		Positive	Negative	Positive rate(%)		Positive	Negative	Positive rate(%)	
Intralymph node PTC metastases	49	48	1	97.96	0.000	44	5	89.80	0.000
Intralymph node ectopic thyroid tissue	15	4	11	26.67		2	13	13.33	

^a^Fisher exact test

### Value of the combined application of Cyclin D1 and P21 in the diagnosis of PTC

The diagnostic efficacy of each immune indicator was compared in Table 5. the Se (93.00%) and NPV (95.04%) of Cyclin D1 were better than P21, while the Sp (99.28%) and PPV (98.89%) of P21 were higher than Cyclin D1. The Se and NPV of P21 and Cyclin D1 independently applied to diagnose PTC were higher than that of the combined application of CK19, HBME-1 and Galectin-3 (Table 5). The combined application of P21 and cyclin D1 had higher diagnostic efficiency. In the differential diagnosis of intralymph node PTC metastases and intralymph node ectopic thyroid tissue, the Se (97.96%) and NPV (91.67%) of Cyclin D1 were better than P21, while the Sp (86.67%) and PPV of P21 (95.65%) were higher than Cyclin D1. The diagnostic efficiency of the two combined applications is the same as that of Cyclin D1 independent applications (Table 5).

**Table 5:** Comparison of the sensitivity, specificity, positive predictive value and negative predictive value of different Biomarker

Diagnosis of PTC primary lesions				
Biomarker	Se	Sp	PPV	NPV
Cyclin D1	93.00%	96.06%	94.41%	95.04%
P21	88.50%	99.28%	98.89%	92.33%
Cyclin D1 + P21	96.00%	95.69%	94.12%	97.09%
CK19 + HBME-1 + Galectin-3	65.00%	99.28%	98.48%	79.83%
Differential diagnosis intralymph node PTC metastases and intralymph node ectopic thyroid tissue				
Biomarker	Se	Sp	PPV	NPV
Cyclin D1	97.96%	73.33%	92.31%	91.67%
P21	89.80%	86.67%	95.65%	72.22%
Cyclin D1 + P21	97.96%	73.33%	92.31%	91.67%

*Se *Sensitivity, *Sp *Specificity, *PPV *Positive predictive value, *NPV *Negative predictive value

## Discussion

The incidence of well-differentiated thyroid cancer has risen sharply over the past two decades and is expected to become the fourth most common cancer worldwide by 2030, with papillary thyroid carcinoma (PTC) being the most common type [1]. When papillary carcinoma lacks typical lesions in histological structure and nuclear morphology, it is very difficult to differentiate it from benign lesions with similar morphology. For example, the follicular variant of papillary carcinoma (FVPTC) is macroscopically similar to encapsulated follicular adenoma, consisting of small- to medium-sized irregularly shaped follicles without papillary glia, and ground-glass-like nuclei may also be present in foci of follicular adenomas. Benign papillary hyperplasia lesions may have branched and complex papillary structures. The cells can appear vacuolated, the nuclei are enlarged and lightly stained, and occasionally, nuclear grooves are seen. Thus, it can be difficult to differentiate from PTC. The tumor cells in the remaining frozen specimens of PTC can be easily stretched and deformed, making histological diagnosis difficult. Papillary microcarcinoma (PTMC) may have only a few follicular lesions, thereby affecting clinical management. Traditional immune markers such as CK19, Galectin-3, and HBME-1 have low sensitivity or specificity for independent diagnosis, making microfocal lesions difficult to identify. In our daily work, we observed that CK19 was strongly expressed in PTC and was also positively expressed in normal thyroid tissue, but the positive intensity was slightly different. Therefore, some studies believe that the value of CK19 in the independent diagnosis of PTC is limited; it is only possible to suggest the existence of PTC when it is strongly positive [6]. Although Galectin-3 has high sensitivity [7], it has poor specificity, as it is expressed in most benign thyroid nodules and chronic lymphocytic thyroiditis (CLT) [8]. In the literature, the positivity rate of HBME-1 in PTC is more than 90% [9], but it also shows positivity in benign nodular thyroid lesions and benign papillary hyperplasia lesions; thus, it also has low specificity [6]. Due to the diagnostic limitations of traditional immune markers, multi-index combined detection is often required to improve the diagnostic accuracy. O'Grady [10] found that when analyzing the high incidence of PTC, researchers focus only on the use of sensitive detection methods, resulting in overdiagnosis in some cases. At the same time, with the continuous discovery of new borderline thyroid lesions and benign and malignant “gray areas”, new immune markers with high sensitivity and specificity are urgently needed for auxiliary diagnosis.

The CCND1 gene was first identified in parathyroid adenomas due to the presence of a centromeric inversion of chromosome fourteen [11]. This defect alters the structure of chromosomes, leading to uncontrolled cell proliferation. Cyclin D1 protein is overexpressed in various types of tumors [12]. The most classic is the specific marker for mantle cell lymphoma (MCL) [13]. In 2015, Lamba Saini [14] first proposed the use of Cyclin D1 as a diagnostic marker for "well-differentiated tumors of uncertain malignant potential (WDT-UMP)" and as a candidate marker for PTC. WDT-UMP is a lesion in follicular adenomatous nodules of the thyroid with focal nuclear features of PTC, which in this study is considered to be a precursor to FVPTC. Cyclin D1 is highly expressed in nuclear characteristic regions with focal PTC but not in surrounding adenomatous regions. However, the study only collected 13 groups of cases, so evidence from large data samples are still needed. Since the concept of WDT-UMP has not been widely accepted, borderline tumors—noninvasive follicular thyroid neoplasm with papillary-like nuclear features (NIFTP)—have been added to the WHO (2017) classification of endocrine tumors to define such lesions. However, Sora Jeon [15] found that the difference between NIFTP and FVPTC lies in the low expression of Cyclin D1b at the mRNA and protein levels. The function of Cyclin D1 in PTC is still unclear; more comprehensive histological sample studies are needed. Previous studies have found that P21 is closely related to tumorigenesis, and in vivo tumor model studies have revealed that P21-deficient mice exhibit sensitivity to hematopoietic, epithelial, and endothelial tumor formation [16]. However, deletion of P21 in a mouse model of prostate cancer resulted in the reduced incidence and invasiveness of prostate cancer [17]. The conflicting results of these studies suggest that P21 has dual roles in tumorigenesis and development, being either oncogenic or tumor suppressive depending on the tumor type and subcellular localization [5].

In our study, various types of histological samples were collected, and it was found that the expression of Cyclin D1 and P21 was localized in the nucleus. The positivity rate in PTC was much higher than that in adjacent tissues and other benign lesions (follicular adenoma and follicular epithelial papillary proliferative lesions), and the sensitivity (93%, 88.5%), specificity (96.06%, 99.28%), positive predictive value (94.41%, 98.89%), and negative predictive value (95.04%, 92.33%) were all high, which support their ability to accurately distinguish PTC from other similar benign lesions. The sensitivity of the combined detection of the two was 96.00%, which was significantly higher than that of the combined detection of the classical antibody markers CK19 + /HBME-1 + /Galectin-3 + (sensitivity 65.00%). The correlation analysis of clinicopathological characteristics showed that the expression of Cyclin D1 and P21 was positively correlated with tumor size and lymph node metastasis (*P* < 0.05), suggesting that Cyclin D1 and P21 may participate in the occurrence and development of PTC, the mechanism of which needs to be further explored. However, there was no correlation with sex, age, number of tumor foci, histological subtype, chronic lymphocytic thyroiditis or TNM stage, indicating that the expression of Cyclin D1 and P21 is not different in classical PTC and FVPTC. Differential expression of Cyclin D1 and P21 in PTC and follicular adenoma may help in differential diagnosis. Whether they can distinguish PTC in chronic lymphocytic thyroiditis from atypical nuclear characteristic lesions in chronic lymphocytic thyroiditis is one of the directions of our continuing research.

Lymph node metastasis is an important indicator of the prognosis of PTC. PTC metastases in cervical lymph nodes usually exhibit only a few follicular structures, and the identification of ectopic thyroid tissue is controversial. Studies have shown that thyroid tissue present in cervical lymph nodes is not always malignant [18]. Therefore, the distinction between malignant and benign thyroid tissue in cervical lymph nodes is extremely important for treatment. Other investigators have proposed different morphological criteria for benign thyroid tissue, including benign thyroid tissue in lymph nodes that does not involve nodal parenchyma or multiple lymph nodes, along with the follicle size, a lack of papillary features of PTC, the absence of nuclear enlargement and crowded nuclei, and a lack of psammoma bodies [19]. However, malignant transformation of ectopic thyroid tissue has also been reported [20]. Therefore, PTC metastases in cervical lymph nodes from ectopic thyroid tissue can be easily misdiagnosed when only morphological diagnosis is used. Molecular testing of BRAF^V600E^, NRAS and KRAS and immunohistochemical markers HBME-1 and Galectin-3 has been helpful for the diagnosis of ectopic thyroid tissue, but these markers cannot clearly prove that the ectopic thyroid tissue is benign [21]. Therefore, there is still a need to discover better markers to improve the diagnostic accuracy. Our study found that the sensitivities of Cyclin D1 and P21 for the identification of intralymph node PTC metastases and intralymph node ectopic thyroid tissue were 97.96% and 89.8%, respectively; the specificities were 73.33% and 86.67%, respectively; the PPVs were 92.31% and 95.65%, respectively; and the predicted values were 91.67% and 72.22%, respectively. The combined detection of Cyclin D1 + /P21 + had a sensitivity of 97.96% and a specificity of 73.33%. This indicates that Cyclin D1 and P21 have important value in the identification of intralymph node PTC metastases and intralymph node ectopic thyroid tissue.

The expression of Cyclin D1 is regulated by multiple factors. The Wnt/β-catenin signaling pathway drives the expression of the downstream key target gene CCND1 and was found to play an important role in the occurrence and development of hepatocellular carcinoma, being necessary for proliferation [22]. Activation of Akt-dependent NF–κB/Cyclin D1 pathway promotes triple-negative breast cancer cell proliferation [23]. GSK3β was involved in the development of cervical squamous cell carcinoma by negatively regulating the nuclear accumulation of Cyclin D1 [24], while p21 and p27 jointly stabilize the Cyclin D1 protein by inhibiting its nuclear export [25]. The importance of Cyclin D1 in tumorigenesis and development and its regulatory mechanisms reflect the potential of CDK inhibitors in therapy. Since p21 has dual functions as a tumor suppressor and oncogene and is regulated by multiple pathways at different transcriptional and translational levels, the expression level of p21 should be strictly controlled [26], and its tumor suppressive or oncogenic function varies in different types of tumors. P21 regulates cell proliferation and metastasis by participating in the PI3K-Akt [27] and c-Myc-P21 [28] signaling pathways. Consequently, the P21 protein expression level can affect the sensitivity of tumors to chemotherapy or radiotherapy. In head and neck squamous cell carcinoma, the overexpression of FXR1 binds and destroys P21 mRNA, destabilizing it and reducing p21 protein expression to promote tumor cell proliferation [29]. In vivo studies have reported that silencing LincRNA [30] or MiRNA [31] leads to upregulation of p21 expression, thereby inhibiting tumor cell apoptosis and proliferation. The role of P21 in tumors is further complicated when P21 is used in combination with other CDK inhibitors. P16 and P21 were found to promote tumor growth by enhancing the chemotaxis of monocyte-derived suppressor cells (Mo-MDSCs) [32]. Injection of the P21-P27 fusion protein into the MCF-7 cell line was reported to induce apoptosis and inhibit proliferation [33]. These studies suggest that P21 plays an important role in tumor therapy. Regulation of the expression of P21 could be used as an adjuvant therapeutic approach for some specific types of tumors to inhibit tumor development or reduce drug resistance. Our study found that Cyclin D1 and P21 were both overexpressed in PTC. Spearman rank correlation analysis showed that there was a significant correlation between these two proteins, indicating that both must maintain proper nuclear accumulation and export and balance in their localization, activity and stability. To prevent and treat the occurrence of tumors, the underlying molecular mechanism should be clarified. At the same time, the results of this work are expected to provide new treatment ideas for clinicians treating patients with advanced thyroid cancer who are not sensitive to I^131^ treatment.

In conclusion, Cyclin D1 and P21 can accurately identify PTC and differentiate follicular adenoma from FVPTC. And it can assist in the diagnosis of intralymph node PTC metastases and intralymph node ectopic thyroid tissue. These markers could therefore serve as molecular targets for the clinical treatment of patients with advanced papillary thyroid cancer.
